# Biology and Fertility Life Table of the Green Aphid *Chaetosiphon Fragaefolli* on Strawberry Cultivars

**DOI:** 10.1673/031.012.2801

**Published:** 2012-02-24

**Authors:** Daniel Bernardi, Mauro Silveira Garcia, Marcos Botton, Dori Edson Nava

**Affiliations:** ^1^ Universidade Federal de Pelotas, Faculdade de Agronomia Eliseu Maciel, Universidade Federal de Pelotas, Caixa Postal 354, CEP: 96010-900, Pelotas-RS, Brazil; ^2^ Universidade Federal de Pelotas, Faculdade de Agronomia Eliseu Maciel, Departamento de Fitossanidade/FAEM/UFPel, Cx. Postal 354, CEP: 96010-900, Pelotas-RS, Brazil; ^3^ Laboratório de Entomologia, Embrapa Uva e Vinho, Cx. Postal 130, CEP: 95700-000, Bento Gonçalves-RS, Brazil; ^4^ Laboratório de Entomologia, Embrapa Clima Temperado, Rodovia Br 396, km 78 Cx. Postal 403, 96001-970, Pelotas-RS, Brazil

**Keywords:** aphids, biological cycle, Fragaria, reproduction rate

## Abstract

Our objective was to study the biology and develop a fertility life table for the aphid *Chaetosiphon fragaefolli* (Cockerell) (Hemiptera: Aphididae) on leaves of strawberry, *Fragaria* × *ananassa*, Duchesne ex Rozier (Rosales: Rosaceae), of the cultivars Albion, Aromas, Camarosa, Camino Real, Diamante, Earlibrite, and Saborosa. This study was conducted under controlled conditions: 25 ± 1 ^°^C, 70 ± 10% RH, and 12:12 L:D . Arenas were set up consisting of leaves inside Petri dishes containing 3% agar. Female aphids obtained after the last nymphal ecdysis were individually placed in arenas for 24 hours. The following biological parameters were evaluated: duration and survival of the nymph stage and of the life cycle (nymph-nymph), daily and total fecundity, and adult longevity. The aphids completed their biological cycle on all of the cultivars. The shortest durations (in days) of the nymphal stage were on the cultivars Camino Real and Camarosa (8.67 and 8.74 days, respectively), and the longest was on Aromas (11.12 days). The lowest survival was on cultivar Aromas (51%) and the highest on Saborosa (96%). When the time to development to the adult stage was compared, the aphids developed fastest (14.63 days) and survival was highest (96%) on cultivar Saborosa. Aphids reared on cultivar Aromas leaves had the longest pre—reproductive period (8.74 days), the greatest longevity (26.88 days), and the longest duration of the life cycle (19.76 days). Based on the fertility life table, cultivars Camarosa and Saborosa were the most favorable for development of *C. fragaefolli*, while Albion and Aromas were the most inadequate for aphid development.

## Introduction

Among the insect pests that affect strawberry plants, the green aphid *Chaetosiphon fragaefolli* (Cockerell) (Hemiptera: Aphididae) is one of the most serious (Rondon et al. 2004; Strand 2008). Damage is caused aphids sucking sap from the plant, which provokes reduction in the production and quality of the fruit, especially when fumagin fungus grows on the honeydew that aphids excrete (Nickel et al. 2005; Cédola and Greco 2010). Besides this direct damage, the green aphid is the principal vector of viruses in this crop (Krczal 1982; Nickel et al. 2005; Rondon et al. 2005; Cédola and Greco 2010).

In Brazil, the main strawberry (*Fragaria* × *ananassa*, Duchesne ex Rozier (Rosales: Rosaceae)) cultivars that are cultivated commercially originate from selection programs conducted in the United States, Chile, and Argentina and are principally the cultivars Aromas, Camarosa, Camino Real, Diamante, Dover, Oso Grande, Sweet Charlie, and Ventana; the first four are the most widely cultivated (Antunes and Reisser Junior 2007; Oliveira et al. 2007). Besides these, new varieties are constantly being introduced and evaluated for their production potential in the various production regions, especially cultivars Camarosa, Camino Real, Aromas, and Diamante (Antunes and Reisser Junior 2007; Oliveira et al. 2007).

The aphid *C. fragaefolli* is an anholocyclic species that lacks a sexual cycle; populations are comprised of viviparous females that produce nymphs through parthenogenesis (Dixon 1985).

Although *C. fragaefolli* is found in all of the main strawberry producing regions, there is little information available concerning the biology of this species and the susceptibility of the various strawberry cultivars to attack by this pest.

In this context, basic information for the establishment of integrated pest management programs (IPMs) in strawberry crops should take into account the reproductive potential of this pest on different cultivars. Here, the biology and reproductive potential of *C. fragaefolli* on seven strawberry cultivars was investigated.

## Materials and Methods

This research was conducted at the Entomology Laboratory of Embrapa Uva e Vinho in a climate chamber maintained at 25 ± 1 ^°^C, 70 ± 10% RH, and 12:12 L:D.

For the biology study, aphids were obtained from an insect rearing facility, where the aphids are kept on ‘Festival’ cultivar strawberry plants cultivated in 3 L polyethylene pots containing soil, planting substrate (Plantmax®), and organic material at a 2:1:1 ratio, kept in a screen cage (2 × 2 × 2 m) covered with an anti—aphid screening (0.64 × 0.20 mm) and kept in a greenhouse. This constituted the stock culture. The *C. fragaefolli* in our experiment were transferred to strawberry leaflets from four—month—old plants of the cultivars Albion, Aromas, Camarosa, Camino Real, Diamante, Earlibrite, and Saborosa. The plants were maintained in a climate chamber as described above.

Strawberry leaflets were collected and placed on a 3 mm layer of 3% agar in Petri dishes (1.3 cm high × 6.5 cm diameter), forming arenas. The tip of the leaf stem was capped with a small piece of wet cotton to help keep the leaf from drying. After each arena was set up, female aphids were transferred from the stock culture. A 4 mm^2^ piece of leaf was removed with the female aphid and transferred with forceps to the arena containing the strawberry leaflet, allowing natural transfer of the aphid to the new leaf. This procedure was repeated every four days in order to maintain fresh plant material for feeding.

After the females were transferred (five females per arena), the Petri plates were placed in the climate chamber. The leaflets were inspected twice per day until the first nymph appeared, after which the females were removed.

Once the nymphs became established (one nymph per arena), the biological factors began to be evaluated, registering the duration and survival of the nymphal stages, the number of instars, daily and total fecundity, adult longevity, life cycle duration (nymph—nymph), and total survival. The number of instars was determined daily with a 10× stereomicroscope by looking for the exuvia that had been shed by the nymphs. The longevity of the insects was calculated using the Weibull model distribution (Sgrillo 1982). The experimental design was fully randomized, with 100 repetitions (arenas) per strawberry cultivar.

The data was used to construct a life table, estimating the mean time between generations (T), the net reproductive rate (Ro), the intrinsic rate of increase (r_m_), and the finite growth rate (λ). The data were submitted to analysis of variance, and the means were compared using a paired *t*— test (α = 0.05) using SAS® (SAS Institute 2007).

**Table 1.**
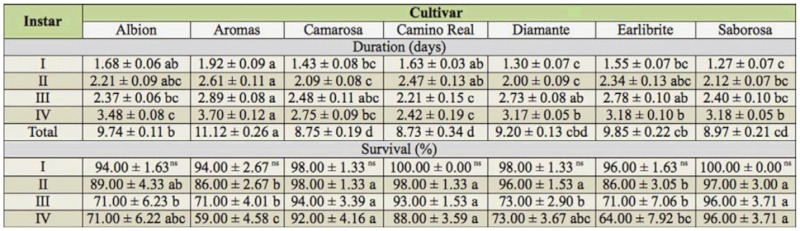
Duration (days) and viability (%) (mean ± SE) of the instars of *Chaetosiphon fragaefolli* on strawberry cultivars in the laboratory.

## Results and Discussion

The green aphid *C. fragaefolli* had four instars on all of the strawberry cultivars. Significant differences were found for various biological parameters of the aphids reared on the different cultivars. Duration of the nymph stage varied significantly (*p* < 0.05), ranging from 8.73 to 11.12 days on cultivars Saborosa and Aromas, respectively (Table 1).

The cultivar Aromas gave the longest nymph development period (11.12 days), differing significantly (*p* < 0.05) from those of cultivars Earlibrite (9.85 days), Albion (9.74 days), Diamante (9.20 days), Saborosa (8.97 days), Camarosa (8.75 days), and Camino Real (8.73 days), which also differed significantly (*p* < 0.05) from each other (Table 1). Similar results were reported by Cédola and Greco (2010); at 25 ^°^C they reported a mean duration of 10.4 days on strawberry cultivars (the cultivars were not specified). The results differed from those reported by Schaefers et al. (1962), who found a nymphal period of 7.4 days for *C.* (*Pentatrichopus*) *fragaefolli* when the aphids were fed on strawberry leaves of the cultivar Shasta.

The fact that cultivar Aromas gave the longest nymph stage interval could be due to antibiosis or antixenosis type resistance. Also, the high mortality rate of the first nymphal instars when reared on cultivar Aromas supports the hypothesis of antibiosis as a defense mechanism of this plant (Smith 2005).

When viability during the nymph phase was examined, cultivar Aromas showed the lowest survival (59%), differing significantly (*p* < 0.05) from Earlibrite (64%), Albion (71%), Diamante (73%), Camino Real (88%), Camarosa (92%), and Saborosa (96%) (Table 1). According to Buchanan et al. (2000), high rates of mortality during this phase may be due to nutritional deficiency of the host, which could be the case for the cultivar Aromas.

Therefore, this high mortality rate found in Aromas suggests the existence of a chemical compound with antinutritional and/or antibiotic characteristics, which could cause a delay or be detrimental to the development of the nymph of *C. fragaefolli* in this cultivar. The influence of allelochemical compounds and their nutritional stage on the biology of this aphid has been reported by Smith (2005); according to the author this is due to low rate of essential nutrients in plant.

**Table 2.**
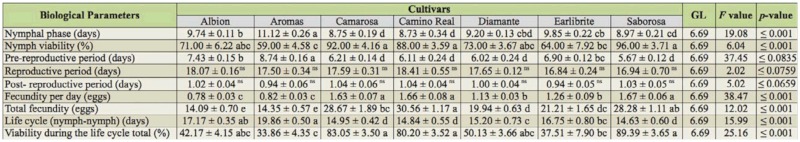
Mean values ± SE of biological parameters of *Chaetosiphon fragaefolli* reared on strawberry cultivars in the laboratory.

**Table 3.**
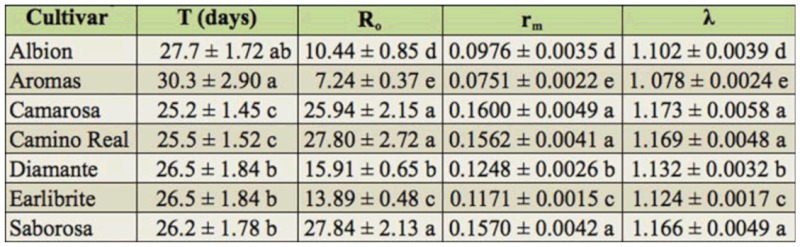
Mean interval between generations (T), net reproductive rate (R_o_), intrinsic growth rate (r_m_), and finite growth rate (λ) of *Chaetosiphon fragaefolli* fed on strawberry cultivar leaves.

In relation to the reproductive phase of *C. fragaefolli*, there was a longer period in the pre—reproductive period (8.74 days) in insects that fed the cultivar Aromas, differing significantly (*p* < 0.05) from other cultivars Albion, Camarosa, Camino Real, Earlibrite, Diamante, and Saborosa (Table 3). According Harrewjin and Minks (1987), the beginning of the reproductive period and, consequently, the production of offspring, is an indication of acceptance of the plant as an ideal host for insect development, which could have caused the differences in our study, mainly in Aromas.

The reproductive period of *C. fragaefolli* was similar for the strawberry cultivars that we evaluated, varying from 16.84 to 18.41 days (Table 2), similar to what was found by Schaefers et al. (1962), who reported a reproductive period of 15.3 days. Cédola and Greco (2010) found a shorter reproductive period (11.8 days) and a longer post reproductive period (1.17 days). A shorter reproductive period was also recorded by Krczal (1982); 4.2 days when *C. fragaefolli* was fed on strawberry cultivar Semperflorens. This difference is possibly due to the food substrate, since according to Awmack and Leather (2002) sucking insects that feed on plant with low levels of soluble nitrogen in the leaves have impaired reproduction and produce few descendants. This could have caused the differences in the duration of the reproductive period between these studies.

The aphids kept in cultivars Aromas and Albion showed low daily and total fecundity of 0.82 and 0.78, respectively (Table 3), differing from all the other cultivars. The results were inferior to those found by Dicker (1952a) and Cédola and Greco (2010), who reported daily fecundities of 2.1 and 2.4 nymphs, respectively, and that reported by Krczal (1982), who found a mean fecundity of 3.3 nymphs (Table 2). According to Awmack and Leather (2002), fecundity is the best biological parameter to demonstrate the quality of a particular host for reproduction of sucking insects.

**Figure 1.**
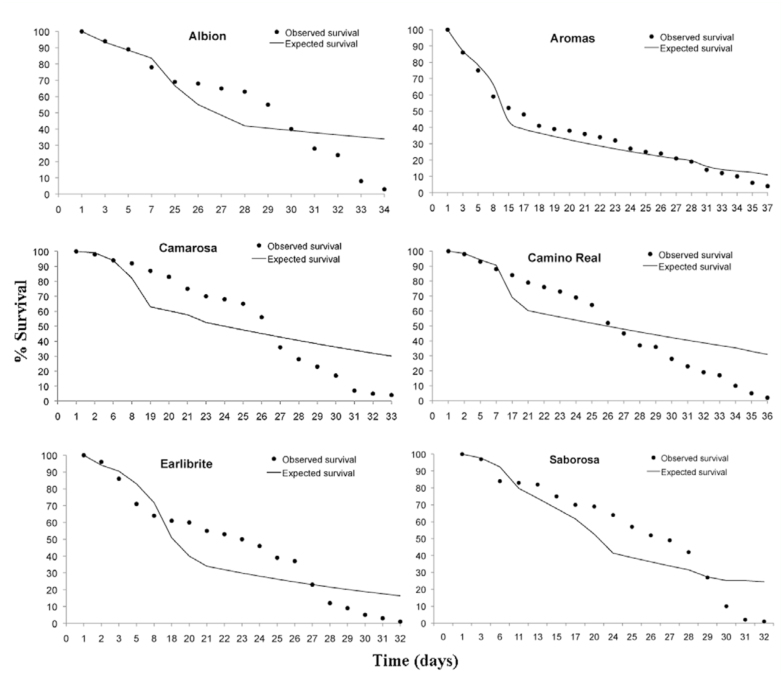
Observed and expected survival (Weibull) of females of *Chaetosiphon fragaefolli* created in strawberry cultivars. Temperature (25 ± 1 ^°^C), relative humidity (70 ± 10%), and photophase (12:12 L:D). The difference between cultivars was five days. High quality figures are available online.

The fact that cultures on Aromas had higher total duration in the nymphal phase, less viability in the total life cycle, and lowest fecundity indicate the occurrence of resistance of antibiosis type in this cultivar. However, it is possible that this cultivar also has feeding non—preference. However, the occurrence of this mechanism has not been investigated in isolation. The high mortality rate in initial phases of the insects makes the hypothesis of the expression of antibiosis the most active mechanism. According to Smith (2005), when a plant is attacked by an insect a reaction occurs in the plant that includes increased protein synthesis and gene expression, that results in activation of a plant defense mechanism. For example, the attacked plant could increase production of chlorophyll or protein to overcome the losses caused by pest attack, triggering the processes of resistance, that may have occurred with cultivar Aromas.

The total life cycle (nymph—nymph) of *C. fragaefolli* also differed among the cultivars; the longest duration and the lowest viability during the period was found on cultivar Aromas, adding approximately five days to the total life cycle duration when compared to aphids fed on cultivars Camarosa, Camino Real, and Saborosa. Analyzing the life table of fertility shows that adults originating from nymphs that had fed on cultivar Aromas had the lowest reproductive performance (Table 3), longest mean interval between generations (T = 30.3 days), lowest intrinsic rate of increase (r_m_ = 0.0751), and a lower finite rate of increase (λ = 1078), differing significantly (*p* < 0.05) from the other cultivars Albion, Camarosa, Camino Real, Diamante, Earlibrite, and Saborosa (Table 3).

The net reproductive rate (R_o_) demonstrated that cultivar Aromas caused a 72% (7.24 times) reduction in female capacity to increase the population in each generation. Among the fertility life table parameters, the value of R_o_ is of great importance to evaluate the quality of the food available to the insect, since this constitutes an innate characteristic of the population (the capacity of each female in a reproductive phase to produce female descendants). This demonstrated the influence of the different genotypes of strawberry on the survival and reproduction of *C. fragaefolli.* It is possible that the negative effects registered in the biological parameters, such as increase nymph development period, reduction in fecundity, and alterations in the reproductive period found for aphids maintained on cultivar Aromas, are related to resistance mechanisms that involve antibiosis or antixenosis, considered a promising tool for use in strawberry pest management. On the other hand, cultivar Camarosa gave the greatest percentage survival during the entire immature phase and for the full life cycle; it was the cultivar that allowed the highest population growth for *C. fragaefolli.*

Aphids fed on cultivar Aromas lived significantly (*p* < 0.05) longer than those fed on cultivars Albion, Camino Real, Diamante, Camarosa, and Earlibrite, which gave similar longevities (Figure 1) close to the 30 days found by Dicker (1952a) when he evaluated the longevity of *C.* (*Pentatrichopus*) *fragaefolli* at 25 ^°^C. Lower values were obtained by Schaefers et al. (1962), Krczal (1982), and Cédola and Greco (2010), who found mean longevities of 21.3, 23.2, and 6.9 days, respectively. It can be seen from the differences in aphid longevity that longevity is depends on the plant host, probably due to differences in the quality of the food ingested. However, it is important to note that the values found in this study were obtained from a plant part, the leaf. This may be one reason that many biological parameters differ from previous studies and corroborate with the studies of Smith (2005) and Smith and Boyko (2006). Smith (2005) suggests the use of leaves as a means of rapid assessment of plant material for aphid resistance because using leaves avoids the potential toxic effects of other plant parts.

When the survival curves of adult *C. fragaefolli* are compared, we can see that at around 20 days of age, the survival of aphids fed on cultivars Camarosa and Saborosa was approximately 85 and 70%, respectively. During this same period, the percent survival of aphids fed on cultivar Aromas was approximately 40% (Figure 1).

Based on the Weibull distribution, there were significant differences in aphid longevity depending on the strawberry cultivar on which they fed, with cultivar Aromas giving the lowest survival compared to the other cultivars (Figure 1).

Overall, results show that some strawberry cultivars affected the development of the green strawberry aphid. The cultivar Aromas stands out, which gave the highest rate of mortality during the immature phase, the lowest fecundity, and the longest life cycle duration, demonstrating resistance against attack by *C. fragaefolli.*

This finding could be of importance for management of this pest species on strawberry, since lower incidence of this pest species would be expected in fields planted with cultivar Aromas. On the other hand, cultivars Camarosa and Saborosa favored the development of *C. fragaefolli.* This fact could provoke differences in management of *C. fragaefolli* by growers, since fields planted with cultivars Camarosa and Saborosa would have a higher rate of population increase of this aphid.

These results suggest that strawberry producers that use the cultivar Aromas will tend to have smaller populations of *C. fragaefolli* due to the greater mortality of the aphids and reduction in the biotic capacity of this pest. On the other hand, if they opt for cultivar Camarosa, the population of this pest species would be expected to increase much faster, possibly requiring differential management.
